# Physical activity during adolescence and risk of colorectal adenoma later in life: results from the Nurses’ Health Study II

**DOI:** 10.1038/s41416-019-0454-1

**Published:** 2019-05-22

**Authors:** Leandro Fórnias Machado de Rezende, Dong Hoon Lee, NaNa Keum, Katharina Nimptsch, Mingyang Song, I-Min Lee, José Eluf-Neto, Shuji Ogino, Charles Fuchs, Jeffrey Meyerhardt, Andrew T. Chan, Walter Willett, Edward Giovannucci, Kana Wu

**Affiliations:** 1 Departamento de Medicina Preventiva, Faculdade de Medicina FMUSP, Universidade de Sao Paulo, Sao Paulo, SP Brazil; 2 Department of Nutrition, Harvard T.H. Chan School of Public Health, Boston, MA USA; 3 Department of Food Science and Biotechnology, Dongguk University, Goyang, South Korea; 4 Molecular Epidemiology Research Group, Max Delbrück Center for Molecular Medicine (MDC), Berlin, Germany; 5 Division of Gastroenterology, Massachusetts General Hospital and Harvard Medical School, Boston, MA USA; 6 Clinical and Translational Epidemiology Unit, Massachusetts General Hospital and Harvard Medical School, Boston, MA USA; 7 Department of Epidemiology, Harvard T.H. Chan School of Public Health, Boston, MA USA; 8 Division of Preventive Medicine, Brigham and Women’s Hospital, Harvard Medical School, Boston, MA USA; 9 Program in MPE Molecular Pathological Epidemiology, Department of Pathology, Brigham and Women’s Hospital and Harvard Medical School, Boston, MA USA; 10 Broad Institute of MIT and Harvard, Cambridge, MA USA; 11 Yale Cancer Center; Department of Medicine, Yale School of Medicine; Smilow Cancer Hospital, New Haven, CT USA; 12 Dana Faber Cancer Institute, Boston, MA USA; 13 Channing Division of Network Medicine, Department of Medicine, Brigham and Women’s Hospital and Harvard Medical School, Boston, MA USA; 14 Department of Immunology and Infectious Diseases, Harvard T.H. Chan School of Public Health, Boston, MA USA

**Keywords:** Cancer epidemiology, Risk factors

## Abstract

**Background:**

Physical activity during adulthood has been consistently associated with lower risk of colorectal cancers, but whether physical activity during adolescence may also play a role in colorectal carcinogenesis is unclear.

**Methods:**

We included 28,250 women in the Nurses’ Health Study II who provided data on physical activity during adolescence (ages 12–22 years) in 1997 and underwent lower bowel endoscopy (1998–2011). We used logistic regression models for clustered data to examine the association between physical activity during adolescence and risk of adenoma later in life.

**Results:**

Physical activity during adolescence was inversely associated with risk of colorectal adenoma (2373 cases), independent of physical activity during adulthood. The multivariable-adjusted odds ratio (OR) of adenoma was 0.89 (95% CI 0.77–1.02; *P*_trend_ = 0.03) comparing women with ≥ 72 metabolic equivalent of tasks-hours/week (MET-h/week) to < 21 MET-h/week. Women with high physical activity during both adolescence (≥53.3 MET-h/week) and adulthood (≥23.1 MET-h/week) had significantly lower risk of adenoma (all adenomas: OR 0.76; 95% CI 0.66–0.88; advanced adenoma: OR 0.61; 95% CI 0.45–0.82) compared to women with low physical activity during both stages of life.

**Conclusions:**

Our findings suggest that physical activity during adolescence may lower the risk of colorectal adenoma later in life.

## Background

Most of the evidence on risk factors for colorectal cancer (CRC) is based on epidemiological studies including mid-to late life populations.^1^ Considering the long process of colorectal carcinogenesis, it is biologically plausible that early-life exposures (e.g., those affecting insulin-IGF pathways) may contribute to CRC risk.^2,3^ The recent rise in CRC incidence rates among adults age < 50 years, (e.g., in the US incidence rates of early-onset CRC increased by 22% between 2000 and 2013) strongly supports that early-life exposures are involved in CRC development.^4^ However, studies examining a role of early-life exposures (other than body fatness) on colorectal carcinogenesis are limited.^5–8^

Physical activity during adulthood is one of the most consistent factors associated with reduced risk of CRC.^1,9,10^ Evidence suggests that physical activity during adulthood may potentially act during early stages of colorectal carcinogenesis by reducing the risk of adenoma, especially advanced adenoma,^11^ an established precursor of CRC.^2,3,12^ Notwithstanding, there is limited evidence regarding early-life physical activity and risk of colorectal adenomas and cancer.^13–15^ To the best of our knowledge, only two case-control studies have examined these associations previously. Findings from these two studies suggested that higher occupational physical activity at age 15 to 19 years was associated with lower risk of CRC.^13,14^ However, potential residual confounding was a major limitation of these studies which did not adjust for potential confounders such as socioeconomic status, dietary factors, smoking, and family history of CRC. Recall bias in case-control studies assessing the association between physical activity and cancer is also a concern.^9^

We hypothesise that high physical activity during adolescence is associated to lower risk of colorectal adenoma later in life, which may have important public health implications for adolescents in terms of cancer prevention. To test this hypothesis, we utilised data from a large cohort study of US women, the Nurses’ Health Study II (NHSII).

## Methods

### Study population

The NHSII enrolled 116,608 female nurses residing in the US aged 25–42 years in 1989, when participants completed a baseline self-administered questionnaire about lifestyle risk factors and diagnosed conditions. Since then, biennial questionnaires were sent to update this information, with response rates over 93%. More details about the NHSII are described elsewhere.^16,17^ The study protocol was approved by the institutional review boards of the Brigham and Women’s Hospital and the Harvard T.H. Chan School of Public Health, and those of participating registries as required.

### Assessment of physical activity

The 1997 questionnaire inquired about physical activity during adolescence and early adulthood. Participants reported average hours a week (none, 1, 2–5, 6–10, 11–20, 21–40, 41–60, 61–90, 90+ h/week) of walking to and from school or work, moderate recreational activities (e.g., hiking, walking for exercise, casual cycling, yard work), and strenuous recreational activities (e.g., running, aerobics, lap swimming) during grades 7–8 (ages 12–13 years), grades 9–12 (ages 14–17 years), ages 18–22, 23–29, and 30–34 years. We assigned average metabolic equivalent of task (MET) for each of these activities to classify intensities (i.e., walking 3 MET, moderate 4.5 MET, and strenuous 7 MET) based on the compendium of physical activities.^18,19^ We summed MET-h/week in each of these activities to obtain total physical activity. The 1997 questionnaire also inquired about the time spent watching television [(TV) (none, 1, 2–5, 6–10, 11–20, 21–40, 41–60, 61–90, ≥ 91 h/week)] during adolescence and early adulthood. For this analysis, we calculated the average of total physical activity (MET-h/week) from ages 12 to 22 years.

Adult recreational physical activity (32–64 years old) was assessed in 1989, 1991, 1997, 2001, 2005, and 2009.^16,20^ Participants reported average time spent per week on a variety of recreational activities and hours per week spent watching TV. We previously showed that in the NHS time spent watching TV in adults predicted risk of type II diabetes better than other measures of sedentary behaviours.^21,22^

We assigned MET values for each of these activities to obtain average total physical activity (in MET-h/week) in each questionnaire cycle where physical activity was assessed.^18,19^ Measures of physical activity have been validated previously (for more detail on reproducibility and validity of the physical activity questionnaires refer to Supplemental Material and our previous publications.^16,17,20,23–26^) Cumulative average adult physical activity was calculated using all available data up to and including the questionnaire 2 years prior to the follow-up cycle at which the most recent endoscopy was reported. Total physical activity during adolescence and cumulative average adult physical activity were weakly correlated (Spearman *r* = 0.19; *P* < 0.001).

### Assessment of dietary factors and other covariates

In 1991 and every 4 years thereafter, diet was assessed through a validated semi-quantitative food frequency questionnaire (FFQ).^27,28^ In addition, in 1998, 47,355 participants (55% of the cohort), at that time 34–51 years old, completed a validated FFQ inquiring about diet during high school.^29^ Previous analyses showed that the risk factor profiles of this subsample were similar to those who did not respond to the high school FFQ.^8^

Height and current weight were obtained on the 1989 baseline questionnaire which also included a 9-level pictogram on body shape to assess body fatness (1 = most lean body shape and 9 = most overweight body shape) at age 5, 10 and 20 years. Weight and other relevant covariates such as lifestyle factors (e.g., aspirin use, smoking status, alcohol intake, family history of CRC) were updated every 2 years.^6^

### Outcome ascertainment

Polyps are often asymptomatic and detected during a lower bowel endoscopy (i.e., either sigmoidoscopy or colonoscopy). Between 1998 and 2011, participants were asked on their biennial follow-up questionnaire whether they underwent a lower bowel endoscopy, the reasons for endoscopy (symptoms or screening) and whether colorectal polyps were diagnosed. Participants who reported a diagnosis of colorectal polyp were mailed a consent form requesting permission to obtain and review their medical records. Study investigators who were blinded to exposure status (e.g., physical activity) reviewed medical records and recorded anatomical location (proximal, distal, and rectum), subtype (adenoma only, serrated lesions only, both adenoma and serrated lesions), and histology and size (advanced: defined as size ≥ 1 cm or any mention of villous histology or high-grade dysplasia; non-advanced: < 1 cm and tubular adenomas) of colorectal polyps. Serrated lesions included the following subtypes: hyperplastic polyp, sessile serrated adenoma/polyp, and traditional serrated adenoma.^30^

### Statistical analysis

For this analysis, we included 28,250 women who responded to a) the 1997 questionnaire, which included information about physical activity during adolescence and adulthood, b) the 1998 FFQ high school questionnaire, and c) underwent at least one lower bowel endoscopy during our follow-up period, i.e., 1998 to 2011. To consider individuals who underwent multiple endoscopies between 1998 and 2011 and reduce potential bias due to time-varying exposure, we used an Anderson-Gill data set structure with a new record for each 2 year follow-up during which participants underwent an endoscopy.^31^ Therefore, participants who underwent multiple endoscopies during follow-up could have multiple observations in the dataset. Exposure and covariates were set at one cycle (2 years) prior the endoscopy. Once a participant was diagnosed with one or more polyps, that participant was censored for all subsequent follow-up cycles.

We used multivariable logistic regression (PROC GENMOD, SAS 9.4, SAS institute Inc., Cary, NC, USA) for clustered data (i.e., each participant was defined as a cluster, therefore accounting for multiple endoscopies) to estimate odds ratios (OR) and 95% confidence intervals for the association between total physical activity during adolescence (<21, 21 to 35.9, 36 to 47.9, 48 to 71.9, ≥ 72 MET-h/week) and risk of adenoma. Categories of physical activity were derived based on its distribution and informative cutoffs.^23^ We also estimated associations per 21 MET-h/week (i.e., equivalent to 1 h of moderate intensity physical activity every day, which is the recommended physical activity level for children and adolescents)^32^ and tested for trend via a Wald test by including the median of physical activity in each category as a single continuous exposure variable into the models.

We ran different multivariable models adjusting for several adolescent and adult covariates selected based on the literature on known or suspected risk factors for colorectal adenomas or cancer.^1,5–8^ The first model (age-adjusted) included age at baseline, time period of endoscopy, number of reported endoscopies, time in years since most recent endoscopy and reason for current endoscopy. The second multivariable model (Model 2) was additionally adjusted for height (continuous), body fatness (1, 2, 3, 4, 5, ≥ 6) at age 5 years (body shape at age 5 was the strongest predictor of adenoma^6^), dietary intake during adolescence [high school FFQ: total calories (quintiles), unprocessed red meat and processed meat (quintiles), total dairy (quintiles), and total fibre (quintiles)], current (adult) aspirin use (≥2 or <2 times/week), current (adult) alcohol intake (< 4.9, 5–9.9, 10–14.9, ≥ 15 g/d), current pack-years of smoking (never, 0–10, > 10–20, > 20–40, > 40 pack-years), and family history of CRC (yes/no). Associations were also examined after further adjustment for cumulative average adult physical activity (quintiles), adult body mass index (BMI, < 25, 25 to 29.9, ≥ 30 kg/m^2^), and time spent watching TV during adolescence (< 3.5, 3.5 to 6.9, 7 to 10.4, 10.5 to 13.9, ≥ 14 h/week). We examined other potential confounders (total folate intake, total calcium intake and western dietary pattern during adolescence, pack-years of smoking before age 20, BMI at age 18, postmenopausal hormone use, total fibre, red and processed meat intake during adulthood) by including these variables separately (i.e., one by one) to Model 2. Adjustment for these variables did not alter the magnitude of associations, therefore, we excluded them from the final model.

To assess interactions, we studied associations after stratification by family history of CRC, age at adenoma diagnosis (<50 years and ≥ 50 years), BMI at 18 years (<23 kg/m^2^ and ≥ 23 kg/m^2^), and smoking status (never and ever). Tests for interaction were performed by including the multiplicative term (cross-product term) of the exposure and each stratification variable in the model and using a Wald test to assess statistical significance.

During adolescence physical activity levels were on average higher (median 40.1 MET-h/week; interquartile range from 23.8 to 70.4) than during adulthood (median 26.4 MET-h/week; interquartile range from 8.7 to 28.6). To assess joint associations of physical activity during adolescence and adulthood with adenoma, we classified participants into four groups according to physical activity and stage of life defining high physical activity as highest tertile (≥53.3 MET-h/week for adolescence and ≥ 23.1 MET-h/week for adulthood) and low physical activity as the bottom two tertiles. Cut-offs were determined post hoc based on our observation that inverse associations between physical activity during age 12–22 years and adenoma were only seen with physical activity levels above 48 to < 72 MET-h/week. We used the highest tertile (≥ 53.3 MET-h/week) to define high physical activity during adolescence. Both subgroup and joint association analyses were adjusted for the same covariates included in Model 2.

We used SAS 9.4 for all analyses (SAS institute Inc., Cary, NC, USA). A two-sided *P* value of 0.05 was considered statistically significant.

## Results

Among 28,250 women included in the study, 2373 adenoma cases were diagnosed between 1998 and 2011 (proximal colon: 1171, distal colon: 1029, rectum: 398). Characteristics of the participants by level of physical activity during adolescence are shown in Table 1. Participants with higher levels of physical activity during adolescence were more likely to have lower body fatness at 5–20 years and lower BMI at 18 years. On the other hand, they were more likely current smokers and to have higher total energy and unprocessed red meat intake during both adolescence and adulthood (Table 1).

**Table 1:** Baseline characteristics of participants by total physical activity during adolescence, Nurses’ Health Study II, 1997

Characteristics^a^	Total physical activity during adolescence (in MET-h/week)				
	< 21 (*n* = 5689)	21 to < 36 (*n* = 6589)	36 to < 48 (*n* = 4568)	48 to < 72 (*n* = 6019)	72+ (*n* = 5385)
Age at 1997 questionnaire return, yrs	51.4 (4.8)	51.0 (4.9)	50.8 (5.0)	50.7 (5.1)	50.5 (5.1)
Number of endoscopies during the study period (*n*)	1.6 (0.8)	1.6 (0.8)	1.5 (0.8)	1.5 (0.8)	1.6 (0.8)
Adult height (inches)	64.8 (2.5)	64.8 (2.6)	65.0 (2.6)	65.0 (2.6)	65.1 (2.7)
BMI at age 18 years (kg/m^2^)	21.4 (3.5)	21.2 (3.2)	21.1 (3.1)	21.0 (3.0)	20.8 (2.8)
Current BMI (kg/m^2^)	25.7 (5.6)	25.6 (5.4)	25.7 (5.7)	25.7 (5.4)	26.0 (5.5)
Waist circumference in 1993 (inches)	30.9 (5.1)	30.8 (5.1)	30.7 (5.0)	30.5 (4.8)	30.7 (5.0)
Hip circumference in 1993 (inches)	39.4 (4.4)	39.4 (4.4)	39.3 (4.3)	39.2 (4.3)	39.3 (4.3)
Waist/hip ratio	0.78 (0.08)	0.78 (0.08)	0.78 (0.08)	0.78 (0.08)	0.78 (0.08)
Body shape^c^ (% of ≥ 5)					
At 5 years of age	8.6	7.1	6.3	6.6	5.0
At 10 years of age	16.5	13.2	11.6	10.9	8.4
At 20 years of age	14.3	10.8	9.6	8.4	6.8
Smoking					
Before 20 years of age (%)	24.4	22.7	22.9	21.7	23.5
Current smokers (%)	5.9	6.7	7.3	8.3	10.0
Current alcohol intake (g/d)	3.9 (6.3)	4.2 (6.5)	4.2 (6.4)	4.1 (6.3)	4.2 (6.5)
Current physical activity (MET-h/week)	17.2 (17.9)	19.0 (18.1)	20.6 (20.5)	22.2 (20.4)	26.0 (24.0)
Time spent watching TV					
During adolescence (hours/week)	7.6 (6.3)	7.5 (6.0)	7.5 (5.8)	7.7 (6.0)	8.1 (6.2)
Current (hours/week)	8.9 (6.9)	8.8 (6.4)	8.8 (6.4)	9.1 (6.6)	9.3 (6.8)
Premenopausal (%)	52.5	52.9	52.2	52.5	52.1
Family history of CRC (%)	27.7	27.4	25.6	26.3	27.9
Current aspirin use (≥ 2 times/week)	8.8	8.7	9.8	9.2	9.4
Dietary intake during adolescence					
Total energy intake (kcal/day)	2609 (778)	2692 (756)	2739 (767)	2797 (775)	2934 (799)
Unprocessed red meat (g/day)	105.3 (49.2)	107.7 (52.2)	107.1 (51.2)	108.3 (51.7)	111.5 (52.7)
Processed meat (g/day)	23.5 (20.1)	22.6 (18.3)	22.7 (17.9)	22.5 (18.1)	23.9 (19.8)
Total dairy (servings/day)	2.6 (1.5)	2.8 (1.4)	2.8 (1.5)	3.0 (1.5)	3.1 (1.6)
Total fibre (g/day)	19.8 (4.9)	20.5 (5.2)	20.8 (5.1)	21.2 (5.3)	21.5 (5.5)
Total calcium (mg/day)	1057 (358)	1078 (344)	1088 (343)	1097 (340)	1091 (342)
Total folate (μg/day)	304 (88)	317 (91)	318 (90)	326 (98)	328 (96)
Adult dietary intake^b^					
Total energy intake (kcal/day)	1707 (474)	1766 (470)	1793 (479)	1817 (489)	1886 (508)
Unprocessed red meat (g/day)	51.8 (40.6)	54.0 (39.3)	55.3 (41.6)	56.1 (42.5)	61.0 (44.7)
Processed meat (g/day)	6.3 (10.0)	6.2 (8.8)	6.6 (9.7)	6.5 (8.6)	7.4 (10.1)
Total fibre (g/day)	18.8 (5.4)	19.2 (5.5)	19.2 (5.3)	19.4 (5.3)	19.2 (5.2)
Total calcium (mg/day)	1072 (433)	1074 (415)	1072 (423)	1064 (420)	1041 (404)
Total folate (μg/day)	473 (236)	480 (230)	478 (228)	482 (235)	474 (224)

For continuous variables mean values and standard deviations are presented

*BMI* body mass index, *MET* metabolic equivalent of tasks

^a^All variables are age-standardised except for age

^b^Cumulative updated average of intake from 1991 and 1995 food frequency questionnaires

^c^9-level figure on body shape; 1 = most lean body shape, 9 = most overweight body shape, i.e., higher values represent higher body fatness

Physical activity during adolescence was inversely associated with risk of adenoma, independent of physical activity during adulthood (Table 2). The magnitude of association was modest, with multivariable-adjusted OR of 0.89 (95% CI 0.77 to 1.02) comparing ≥ 72 MET-h/week to < 21 MET-h/week (reference group). The OR of adenoma per 21 MET-h/week was 0.96 (95% CI 0.93 to 0.99; *P*_trend_ = 0.02). Results were similar after further adjustment for physical activity and BMI during adulthood and TV watching during adolescence (Supplementary File, Table S1).

**Table 2:** Odds ratio of colorectal adenoma according to total physical activity during adolescence by location of adenoma. Nurses’ Health Study II, 1997–2011

	Total physical activity during adolescence (in MET-h/week)						
	<21	21 to < 36	36 to < 48	48 to < 72	72+	Per 21 MET-h/week	*P*_trend_
All adenomas							
*N* Cases	503	579	416	466	409		
Age-adjusted^a^	1	1.01 (0.90–1.15)	1.06 (0.93–1.22)	0.90 (0.79–1.03)	0.88 (0.77–1.01)	0.96 (0.93–0.99)	0.01
Multivariable^b^	1	1.02 (0.90–1.16)	1.07 (0.94–1.23)	0.91 (0.80–1.04)	0.89 (0.77–1.02)	0.96 (0.93–0.99)	0.02
Multivariable^b^ plus adult physical activity	1	1.02 (0.90–1.16)	1.08 (0.94–1.23)	0.92 (0.80–1.05)	0.90 (0.78–1.03)	0.96 (0.93–1.00)	0.03
Location of adenoma							
Proximal adenoma							
*N* Cases	252	290	205	227	197		
Age-adjusted^a^	1	1.02 (0.85–1.21)	1.05 (0.87–1.27)	0.88 (0.73–1.06)	0.85 (0.70–1.03)	0.95 (0.91–0.99)	0.03
Multivariable^b^	1	1.02 (0.85–1.21)	1.06 (0.87–1.28)	0.89 (0.73–1.07)	0.86 (0.70–1.04)	0.95 (0.91–1.00)	0.04
Multivariable^b^ plus adult physical activity	1	1.02 (0.86–1.22)	1.06 (0.88–1.29)	0.90 (0.74–1.08)	0.87 (0.72–1.07)	0.96 (0.91–1.00)	0.07
Distal adenoma							
*N* Cases	219	255	174	193	188		
Age-adjusted^a^	1	1.03 (0.85–1.23)	1.02 (0.83–1.25)	0.86 (0.71–1.05)	0.93 (0.76–1.14)	0.97 (0.92–1.02)	0.21
Multivariable^b^	1	1.04 (0.87–1.26)	1.04 (0.85–1.28)	0.88 (0.72–1.07)	0.94 (0.76–1.15)	0.97 (0.92–1.02)	0.22
Multivariable^b^ plus adult physical activity	1	1.04 (0.86–1.26)	1.04 (0.85–1.28)	0.88 (0.72–1.08)	0.94 (0.77–1.16)	0.97 (0.92–1.02)	0.26
Rectal adenoma							
*N* cases	84	86	73	81	74		
Age-adjusted^a^	1	0.90 (0.66–1.21)	1.11 (0.80–1.52)	0.93 (0.68–1.27)	0.94 (0.69–1.30)	0.99 (0.92–1.07)	0.80
Multivariable^b^	1	0.91 (0.67–1.24)	1.13 (0.82–1.55)	0.94 (0.69–1.29)	0.94 (0.68–1.30)	0.99 (0.91–1.07)	0.76
Multivariable^b^ plus adult physical activity	1	0.92 (0.68–1.25)	1.15 (0.84–1.58)	0.96 (0.70–1.32)	0.97 (0.69–1.35)	0.99 (0.92–1.08)	0.90

^a^Adjusted for age, time period of endoscopy, number of reported endoscopies, time in years since most recent endoscopy and reason for current endoscopy

^b^Additionally adjusted for height (inches), body shape at 5 years (1, 2, 3, 4, 5, ≥ 6), dietary intake during adolescence [from high school FFQ: total calories (quintiles), unprocessed red meat and processed meat (quintiles), total dairy (quintiles), and total fibre (quintiles)], current (adult) aspirin use (≥2 or <2 times/week), current (adult) alcohol intake (<4.9, 5–9.9, 10–14.9, 15 + g/d), current (adult) pack-years of smoking (never, 0–10, > 10–20, > 20–40, 40 + pack-years), and family history of CRC (yes/no)

When we analysed data separately by location of adenoma, physical activity during adolescence was associated with lower risk of proximal adenoma, but not rectal or distal adenomas (Table 2). Furthermore, physical activity during adolescence was not associated with risk of serrated lesions only (Table 3). The inverse associations also appeared to be stronger among women with no family history of CRC and age at adenoma diagnosis ≥ 50 years, although the interaction was only statistically significant for age at diagnosis (*P* < 0.01) (Table S2).

**Table 3:** Odds ratio of colorectal polyp according to total physical activity during adolescence by stage of adenoma and subtype of colorectal polyp. Nurses’ Health Study II, 1997–2011

	Total physical activity during adolescence (in MET-h/week)						
	< 21	21 to < 36	36 to < 48	48 to < 72	72+	Per 21 MET-h/week	*P*_trend_
Colorectal adenoma by stage							
Advanced							
*N* Cases	105	156	110	106	89		
Age-adjusted^a^	1	1.31 (1.02–1.69)	1.34 (1.02–1.76)	0.98 (0.75–1.29)	0.92 (0.69–1.23)	0.94 (0.88–1.00)	0.07
Multivariable^b^	1	1.34 (1.04–1.73)	1.36 (1.03–1.78)	1.00 (0.76–1.32)	0.91 (0.68–1.23)	0.94 (0.88–1.00)	0.06
Multivariable^b^ plus adult physical activity	1	1.35 (1.05–1.74)	1.38 (1.05–1.81)	1.02 (0.77–1.35)	0.94 (0.70–1.27)	0.95 (0.88–1.01)	0.11
Non-advanced							
*N* Cases	294	318	225	257	247		
Age-adjusted^a^	1	0.95 (0.81–1.12)	0.98 (0.82–1.17)	0.85 (0.71–1.01)	0.90 (0.76–1.07)	0.97 (0.93–1.01)	0.16
Multivariable^b^	1	0.96 (0.81–1.13)	0.99 (0.83–1.19)	0.86 (0.72–1.02)	0.91 (0.76–1.09)	0.97 (0.93–1.02)	0.21
Multivariable^b^ plus adult physical activity	1	0.96 (0.81–1.13)	0.99 (0.83–1.19)	0.86 (0.72–1.03)	0.92 (0.77–1.10)	0.97 (0.93–1.02)	0.24
Colorectal polyp by subtype							
Adenoma only							
*N* Cases	406	490	341	363	331		
Age-adjusted^a^	1	1.06 (0.93–1.22)	1.08 (0.93–1.26)	0.87 (0.75–1.01)	0.88 (0.76–1.03)	0.95 (0.92–0.99)	0.01
Multivariable^b^	1	1.07 (0.94–1.23)	1.10 (0.95–1.28)	0.88 (0.76–1.02)	0.90 (0.77–1.05)	0.95 (0.92–0.99)	0.01
Multivariable^b^ plus adult physical activity	1	1.07 (0.94–1.23)	1.10 (0.95–1.28)	0.89 (0.76–1.03)	0.90 (0.77–1.06)	0.96 (0.92–0.99)	0.02
Serrated lesions only							
*N* Cases	339	378	272	347	309		
Age-adjusted^a^	1	0.98 (0.84–1.14)	1.02 (0.87–1.21)	0.99 (0.85–1.16)	0.98 (0.83–1.15)	1.00 (0.96–1.04)	0.85
Multivariable^b^	1	0.99 (0.85–1.16)	1.04 (0.88–1.23)	1.01 (0.86–1.18)	0.97 (0.82–1.14)	0.99 (0.95–1.03)	0.69
Multivariable^b^ plus adult physical activity	1	0.99 (0.85–1.15)	1.04 (0.88–1.23)	1.01 (0.86–1.18)	0.97 (0.83–1.15)	0.99 (0.96–1.03)	0.77
Both adenoma and serrated lesions							
*N* Cases	97	89	75	103	78		
Age-adjusted^a^	1	0.81 (0.61–1.09)	1.01 (0.74–1.36)	1.04 (0.79–1.38)	0.89 (0.65–1.20)	1.00 (0.93–1.07)	0.91
Multivariable^b^	1	0.81 (0.61–1.09)	0.99 (0.72–1.35)	1.03 (0.77–1.37)	0.84 (0.61–1.14)	0.98 (0.91–1.06)	0.60
Multivariable^b^ plus adult physical activity	1	0.82 (0.61–1.10)	1.00 (0.73–1.36)	1.05 (0.79–1.40)	0.86 (0.63–1.17)	0.99 (0.92–1.06)	0.74

^a^Adjusted for age, time period of endoscopy, number of reported endoscopies, time in years since most recent endoscopy and reason for current endoscopy ^b^Additionally adjusted for height (inches), body shape at age 5 years (1, 2, 3, 4, 5, ≥ 6), dietary intake during adolescence [from high school FFQ: total calories (quintiles), unprocessed red meat and processed meat (quintiles), total dairy (quintiles), and total fibre (quintiles)], current (adult) aspirin use (≥2 or <2 times/week), current (adult) alcohol intake (< 4.9, 5–9.9, 10–14.9, 15 + g/d), current (adult) pack-years of smoking (never, 0–10, > 10–20, > 20–40, 40 + pack-years), and time spent watching TV during adolescence (0.5, 0.5–1, 1–1.5, 1.5–2, 2 + h/day)

Serrated lesions included the following subtypes: hyperplastic polyp, sessile serrated adenoma/polyp, and traditional serrated adenoma

**Fig. 1:**
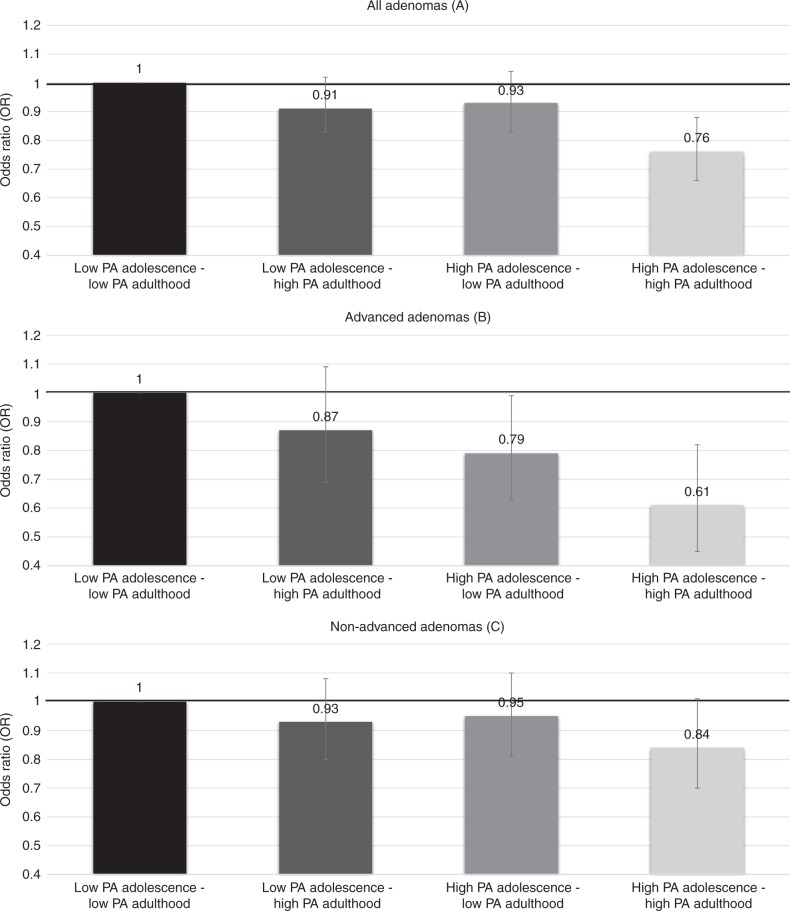
Joint associations of total physical activity during adolescence and adulthood with colorectal adenoma. Nurses’ Health Study II, 1997–2011. *Footnote*: High physical activity (PA) at adolescence was defined as the highest tertile (≥53.3 MET-h/week); low was defined as the two bottom tertiles (<53.5 MET-h/week). High PA at adulthood was defined as highest tertile (≥23.1 MET-h/week); low was defined as two bottom tertiles (<23.1 MET-h/week). Number of all adenomas: *Low PA adolescence- Low PA adulthood* (*n* = 1192); *Low PA adolescence- High PA adulthood* (*n* = 453); *High PA adolescence- Low PA adulthood* (*n* = 463); *High PA adolescence- High PA adulthood* (*n* = 265); Number of non-advanced adenomas: *Low PA adolescence- Low PA adulthood* (*n* = 659); *Low-high* (*n* = 258); *High-low* (*n* = 261); *High-high* (*n* = 163); Number of advanced adenomas: *Low PA adolescence- Low PA adulthood* (*n* = 305); *Low PA adolescence- High PA adulthood* (*n* = 105); *High PA adolescence- Low PA adulthood* (*n* = 103); *High PA adolescence- High PA adulthood* (*n* = 53); Adjusted for age, time period of endoscopy, number of reported endoscopies, time in years since most recent endoscopy and reason for current endoscopy, height (inches), body shape at age 5 years (1, 2, 3, 4, 5, ≥ 6), dietary intake during adolescence [from high school FFQ: total calories (quintiles), unprocessed red meat and processed meat (quintiles), total dairy (quintiles), and total fibre (quintiles)], current (adult) aspirin use (≥2 or <2 times/week), current (adult) alcohol intake (<4.9, 5–9.9, 10–14.9, 15 + g/d), current (adult) pack-years of smoking (never, 0–10, > 10–20, > 20–40, 40 + pack-years), and family history of colorectal cancer (yes/no)

We assessed joint associations of physical activity during adolescence and adulthood with adenoma (Fig. 1, Table S3). Compared to women with low physical activity during both adolescence and adulthood, a lower risk of adenoma was observed for women with high physical activity during adolescence only (OR 0.93; 95% CI 0.83 to 1.04) and during adulthood only (OR 0.91; 95% CI 0.82–1.02), but associations did not reach statistical significance. The strongest inverse association was found for women with high physical activity during both adolescence and adulthood (OR 0.76; 95% CI 0.66 to 0.88, Fig. 1A). When we examined associations by stage of adenoma, inverse associations appeared to be stronger for advanced adenoma than for non-advanced adenoma. Compared to participants with low physical activity during both stages of life, those with high physical activity during both adolescence and adulthood had a 39% lower risk of advanced adenoma (0.61; 95% CI 0.45 to 0.82, Fig. 1B). For non-advanced adenoma, comparing the consistently high to the consistently low physical activity group the OR was 0.84 (95% CI 0.70 to 1.01, Fig. 1C).

## Discussion

In this large study, physical activity during adolescence (ages 12–22 years) was associated with lower risk of colorectal adenomas, independent of physical activity during adulthood. When we examined joint associations of physical activity during adolescence and adulthood with adenoma, we also found that women who were physically active during both adolescence and adulthood had the lowest risk of colorectal adenoma compared to women who were less active in both or one stage of life.

The recent rise in early-onset CRC indicates that early-life exposures may be involved, but the causes underlying these increases have not been elucidated yet.^4^ Our subgroup analyses suggested that physical activity during adolescence was associated with lower risk of adenoma in women diagnosed age ≥ 50 years, but no association was found for adenoma diagnosed under age 50 years or in women with a positive family history of CRC. Individuals with a family history of CRC are generally recommended to start screening at an earlier age than those at average risk for CRC (e.g., in the US average risk individuals are recommended to start screening for CRC at age 50 years).^33^ CRCs among individuals with a family history of CRC, may also have a stronger genetic component and different aetiology than sporadic CRC.^34^ Nonetheless, considering the recent rise of early onset CRC in countries such as the US, these observations warrant further examination in a separate study focusing on the etiology of early onset CRC neoplasia.

Physical activity may potentially affect carcinogenesis by decreasing body fatness, inflammation, and insulin levels.^35–37^ High levels of insulin and insulin resistance, which can stimulate cell proliferation and inhibit apoptosis,^38^ are associated with higher risk of adenoma in adults.^39^ Physical activity throughout life, including during adolescence, may decrease insulin resistance and increase glucose uptake by skeletal muscle,^35,36^ especially by improving body composition (i.e., reducing visceral adipose tissue).^40^ In addition, recent data suggest that physical activity may affect composition and diversity of gut microbiota resulting in more favourable metabolic and inflammatory profiles.^41–43^ However, more human studies on that topic are needed, especially studies accounting for possible confounding by diet.

In adults, physical activity is considered an established protective factor for colon cancer but not rectal cancer.^1,9,10^ Our results suggested that associations may be slightly stronger for adenomas located in the proximal colon, corroborating evidence that associations between physical activity and CRC may differ by sub-sites.^9^ However, recent meta-analyses that examined adult physical activity and CRC by sub-sites suggested that while associations differ for colon vs. rectum they may not differ by sub-sites within the colon i.e., proximal vs. distal.^44,45^

The association between physical activity during adulthood and colorectal adenoma supports a potential protective effect of physical activity on earlier stages of carcinogenesis. A meta-analysis including 20 case-control and cohort studies found a 16% (RR 0.84; 95% CI 0.77 to 0.90) lower risk of colon adenoma among individuals in the highest category of physical activity during adulthood when compared to those in the lowest category of physical activity.^11^ Nonetheless, studies examining the association between early-life physical activity and cancer risk, including colorectal adenoma and CRC, are sparse.^5^ To the best of our knowledge, only two case-control studies investigated the association between early-life physical activity and CRC later in life. A hospital-based case-control study conducted in Italy, including 1225 cases and 4154 controls, found that higher levels of occupational physical activity, but not leisure activity at ages 15 to 19 years was associated with lower risk of colon cancer.^15^ Similarly, another hospital-based case-control study conducted between 1992 and 1997 in the Swiss canton of Vaud also observed an inverse association between occupational physical activity, but not leisure activity at ages 15 to 19 years and risk of CRC.^14^

To our knowledge, our study is the first large prospective analysis to show an inverse association between physical activity during both adolescence and adulthood on risk of colorectal adenoma. When we examined joint associations of physical activity during adolescence and adulthood, we observed that participants with consistently high levels of physical activity during both adolescence and adulthood had the lowest risk of colorectal adenoma compared to those with low levels of physical activity in both or either periods. These associations were slightly stronger for advanced adenoma, a subtype more likely to progress to CRC than small or non-advanced adenoma.^11^ Similarly, in the Harvard Alumni Health Study, a cohort study including 17 148 adults, physical activity was assessed in 1962/1966, when participants were 30 to 79 years of age, and again 1977 (ages 45 to 94 years).^13^ In that study, only high levels of physical activity during both periods, but not in either one, was associated with lower risk of colon cancer. Despite differences in age group, results from the Harvard Alumni study are in accordance with our findings suggesting a possible role of physical activity throughout life on colon carcinogenesis.

Some limitations should be considered while interpreting our results. Physical activity during both adolescence and adulthood was self-reported and some misclassification of exposure is inevitable. However, both adolescent and adult physical activity questionnaires showed reasonable reproducibility, and the adult questionnaire was also validated.^20,24–26^ Additionally, physical activity during adolescence and adulthood were only weakly correlated (*r* = 0.19). Misclassification of physical activity is likely to be non-differential because information was collected prior to diagnosis of colorectal adenoma, and therefore would bias associations towards the null. Although our analyses considered a large number of potential lifestyle and dietary confounders during both adolescence and adulthood, residual confounding due to imperfect adjustment or unmeasured confounders cannot be ruled out.

In conclusion, we found an inverse association between physical activity during adolescence and risk of colorectal adenoma in women, independent of physical activity during adulthood. Participants with consistently high levels of physical activity during both adolescence and adulthood had the lowest risk of colorectal adenoma compared to those with low levels of physical activity during both or either stages of life. Our findings need to be confirmed in other studies but suggest that physical activity during adolescence may play a role in early stages of colorectal carcinogenesis, which may have important implications for cancer prevention.

## Supplementary information

Supplementary material

## Data Availability

Further information including the procedures to obtain and access data from the Nurses' Health Study and Health Professionals Follow-up Study is described at https://www.nurseshealthstudy.org/researchers (email: nhsaccess@channing.harvard.edu) and https://sites.sph.harvard.edu/hpfs/for-collaborators.
