# Supplementary material for: Physical activity during adolescence and risk of colorectal adenoma later in life: results from the Nurses’ Health Study II
Source: Br J Cancer. 2019 May 22;121(1):86–94. doi: 10.1038/s41416-019-0454-1 (PMC6738055; doi:10.1038/s41416-019-0454-1)
Supplement: Supplementary file 1 — Supplementary material [file 41416_2019_454_MOESM1_ESM.docx]

**SUPPLEMENTARY FILE**

**Reproducibility and validity of physical activity questionnaires**

In 2000, a sample of 160 participants that completed the 1997 questionnaire on physical activity was asked about again their average of physical activity during adolescence and early adulthood for sake of examine reproducibility. On average, the correlations between the 1997 and the 2000 assessment was 0.64 for total physical activity, 0.76 for strenuous, 0.70 for strenuous plus moderate. By age group, spearman rho correlation for hours/week in strenuous activity was 0.63 in grade 7 to 8, 0.71 in grades 9 to 12, and 0.69 at ages 18 to 22. Walking activity presented similar reproducibility results, whereas moderate physical activity was lower across all periods (0.37 for grades 7 and 8, 0.36 for grades 9 to 12, and 0.52 for ages 18 to 22).[1]

In adults, physical activity questionnaire showed good validity when comparing physical activity in the previous year to both recall of physical activity in the previous week (r = 0.79) and activity in diaries (r = 0.62). Similar physical activity questions has also showed a have a moderate correlation with maximal oxygen consumption (*r* = 0.54) [2] and lower resting pulse (*r* = 0.45).[3]

**REFERENCES**

1. Baer HJ, Schnitt SJ, Connolly JL, et al. Early life factors and incidence of proliferative benign breast disease. *Cancer Epidemiol Biomarkers Prev*. **14**, 2889-2897 (2005).

2. Jacobs DR, Jr., Ainsworth BE, Hartman TJ, et al. A simultaneous evaluation of 10 commonly used physical activity questionnaires. *Med Sci Sports Exerc*. **25**, 81-91 (1993).

3. Chasan-Taber S, Rimm EB, Stampfer MJ, et al. Reproducibility and validity of a self-administered physical activity questionnaire for male health professionals. *Epidemiology*. **7**, 81-86 (1996).

| **Table S1.** Odds ratio of colorectal polyps according to total physical activity during adolescence by stage of adenoma, location, and subtype of colorectal polyp. Nurses' Health Study II, 1997-2011 | | | | | | | |
| --- | --- | --- | --- | --- | --- | --- | --- |
|  | **Total physical activity during adolescence (in MET-h/week)** | | | | | | |
|  | **<21** | **21 - <36** | **36 - <48** | **48 - <72** | **72+** | **Per 21**  **MET-h/week** | **P_trend_** |
| **All adenomas** |  |  |  |  |  |  |  |
| Multivariable† plus adult PA and BMI, and television watching during adolescence | 1 | 1.03(0.91-1.17) | 1.07(0.94-1.23) | 0.91(0.80-1.05) | 0.88(0.77-1.02) | 0.96(0.93-0.99) | 0.01 |
| Multivariable† plus adult BMI | 1 | 1.02(0.90-1.16) | 1.07(0.93-1.22) | 0.90(0.79-1.03) | 0.88(0.76-1.01) | 0.96(0.92-0.99) | 0.01 |
| Multivariable† plus adult PA and BMI | 1 | 1.02(0.90-1.16) | 1.07(0.93-1.23) | 0.91(0.79-1.04) | 0.88(0.77-1.01) | 0.96(0.93-0.99) | 0.01 |
| **By Location** |  |  |  |  |  |  |  |
| *Proximal adenomas* |  |  |  |  |  |  |  |
| Multivariable† plus adult PA and BMI, and television watching during adolescence | 1 | 1.02(0.86-1.22) | 1.06(0.87-1.28) | 0.89(0.74-1.08) | 0.86(0.70-1.05) | 0.95(0.90-1.00) | 0.04 |
| Multivariable† plus adult BMI | 1 | 1.01(0.85-1.21) | 1.05(0.87-1.27) | 0.88(0.73-1.06) | 0.85(0.69-1.03) | 0.95(0.90-0.99) | 0.03 |
| Multivariable† plus adult PA and BMI | 1 | 1.02(0.85-1.21) | 1.05(0.87-1.28) | 0.89(0.73-1.07) | 0.86(0.70-1.05) | 0.95(0.90-1.00) | 0.04 |
| *Distal adenomas* |  |  |  |  |  |  |  |
| Multivariable† plus adult PA and BMI, and television watching during adolescence | 1 | 1.05(0.87-1.27) | 1.03(0.84-1.27) | 0.88(0.71-1.07) | 0.92(0.74-1.13) | 0.96(0.91-1.01) | 0.14 |
| Multivariable† plus adult BMI | 1 | 1.04(0.86-1.25) | 1.04(0.84-1.27) | 0.87(0.71-1.06) | 0.92(0.75-1.13) | 0.97(0.92-1.02) | 0.17 |
| Multivariable† plus adult PA and BMI | 1 | 1.04(0.86-1.25) | 1.03(0.84-1.27) | 0.87(0.71-1.06) | 0.92(0.75-1.13) | 0.97(0.92-1.02) | 0.18 |
| *Rectal adenomas* |  |  |  |  |  |  |  |
| Multivariable† plus adult PA and BMI, and television watching during adolescence | 1 | 0.93(0.68-1.27) | 1.19(0.86-1.64) | 0.98(0.71-1.35) | 0.97(0.69-1.36) | 0.99(0.92-1.08) | 0.89 |
| Multivariable† plus adult BMI | 1 | 0.91(0.67-1.24) | 1.13(0.82-1.55) | 0.94(0.69-1.29) | 0.94(0.67-1.30) | 0.99(0.91-1.07) | 0.74 |
| Multivariable† plus adult PA and BMI | 1 | 0.92(0.68-1.25) | 1.15(0.83-1.58) | 0.96(0.70-1.32) | 0.96(0.69-1.35) | 0.99(0.92-1.08) | 0.88 |
| **By Stage** |  |  |  |  |  |  |  |
| *Advanced* |  |  |  |  |  |  |  |
| Multivariable† plus adult PA and BMI, and television watching during adolescence | 1 | 1.36(1.05-1.75) | 1.37(1.04-1.81) | 1.02(0.78-1.35) | 0.94(0.70-1.26) | 0.94(0.88-1.01) | 0.09 |
| Multivariable† plus adult BMI | 1 | 1.34(1.04-1.72) | 1.35(1.03-1.78) | 0.99(0.76-1.31) | 0.90(0.67-1.21) | 0.94(0.88-1.00) | 0.05 |
| Multivariable† plus adult PA and BMI | 1 | 1.35(1.05-1.74) | 1.37(1.04-1.80) | 1.01(0.77-1.34) | 0.93(0.69-1.25) | 0.94(0.88-1.01) | 0.08 |
| *Non-advanced* |  |  |  |  |  |  |  |
| Multivariable† plus adult PA and BMI, and television watching during adolescence | 1 | 0.96(0.81-1.13) | 0.99(0.82-1.19) | 0.86(0.72-1.02) | 0.90(0.75-1.08) | 0.97(0.92-1.01) | 0.16 |
| Multivariable† plus adult BMI | 1 | 0.95(0.81-1.12) | 0.99(0.82-1.18) | 0.85(0.71-1.02) | 0.90(0.75-1.08) | 0.97(0.93-1.01) | 0.17 |
| Multivariable† plus adult PA and BMI | 1 | 0.95(0.81-1.12) | 0.99(0.82-1.18) | 0.85(0.71-1.02) | 0.90(0.75-1.08) | 0.97(0.93-1.01) | 0.18 |
| **By subtype** |  |  |  |  |  |  |  |
| *Adenoma only* |  |  |  |  |  |  |  |
| Multivariable† plus adult PA and BMI, and television watching during adolescence | 1 | 1.08(0.94-1.24) | 1.10(0.95-1.29) | 0.89(0.76-1.03) | 0.90(0.77-1.05) | 0.95(0.92-0.99) | 0.01 |
| Multivariable† plus adult BMI | 1 | 1.07(0.93-1.23) | 1.10(0.95-1.28) | 0.88(0.76-1.02) | 0.89(0.76-1.04) | 0.95(0.92-0.99) | 0.01 |
| Multivariable† plus adult PA and BMI | 1 | 1.07(0.93-1.23) | 1.10(0.95-1.28) | 0.88(0.76-1.02) | 0.90(0.77-1.05) | 0.95(0.92-0.99) | 0.02 |
| *Serrated lesions only* |  |  |  |  |  |  |  |
| Multivariable† plus adult PA and BMI, and television watching during adolescence | 1 | 0.98(0.84-1.14) | 1.03(0.88-1.22) | 0.99(0.85-1.16) | 0.94(0.79-1.11) | 0.98(0.95-1.02) | 0.44 |
| Multivariable† plus adult BMI | 1 | 0.99(0.85-1.15) | 1.03(0.88-1.22) | 0.99(0.85-1.16) | 0.95(0.80-1.11) | 0.99(0.95-1.03) | 0.49 |
| Multivariable† plus adult PA and BMI | 1 | 0.98(0.84-1.14) | 1.03(0.87-1.21) | 0.99(0.84-1.15) | 0.94(0.80-1.11) | 0.98(0.95-1.02) | 0.44 |
| *Both adenoma and Serrated lesions* |  |  |  |  |  |  |  |
| Multivariable† plus adult PA and BMI, and television watching during adolescence | 1 | 0.81(0.60-1.09) | 0.98(0.71-1.34) | 1.03(0.77-1.38) | 0.81(0.59-1.12) | 0.97(0.90-1.05) | 0.50 |
| Multivariable† plus adult BMI | 1 | 0.81(0.60-1.08) | 0.97(0.71-1.32) | 1.01(0.76-1.35) | 0.81(0.59-1.10) | 0.97(0.90-1.05) | 0.45 |
| Multivariable† plus adult PA and BMI | 1 | 0.81(0.60-1.09) | 0.98(0.72-1.33) | 1.02(0.76-1.35) | 0.81(0.59-1.11) | 0.97(0.90-1.05) | 0.48 |
| *Adjusted for age, time period of endoscopy, number of reported endoscopies, time in years since most recent endoscopy and reason for current endoscopy. | | | | | | | |
| † Additionally adjusted for height (inches), body shape at age 5 years (1, 2, 3, 4, 5, ≥ 6), dietary intake during adolescence [from high school FFQ: total calories (quintiles), unprocessed red meat and processed meat (quintiles), total dairy (quintiles), and total fiber (quintiles)], current (adult) aspirin use (≥2 or <2 times/week), current (adult) alcohol intake ( < 4.9, 5–9.9, 10–14.9, 15 + g/d), current (adult) pack-years of smoking (never, 0–10, > 10–20, > 20–40, 40 + pack-years), and time spent watching TV during adolescence (0.5, 0.5–1, 1–1.5, 1.5–2, 2 + h/day)  Serrated lesions included the following: hyperplastic polyp, sessile serrated adenoma/polyp, and traditional serrated adenoma. | | | | | | | |

| **Table S2.** Subgroup analysis: Association between total physical activity during adolescence and risk of colorectal adenoma by family history, age at diagnosis, body mass index and smoking. Nurses' Health Study II, 1997-2011 | | | |
| --- | --- | --- | --- |
|  | **Total physical activity during adolescence**  **(Per 21 MET-h/week)** | | |
|  | **Odds ratio** | **95% Confidence interval** | **P_interaction_** |
| **All adenomas** |  |  |  |
| **Family history of CRC** |  |  | 0.14 |
| No (N cases = 1807) | 0.95 | (0.91 to 0.98) |  |
| Yes (N cases = 564) | 1.01 | (0.94 to 1.08) |  |
| **Age at diagnosis** |  |  | <0.01 |
| <50 years (N cases = 538) | 1.01 | (0.94 to 1.08) |  |
| ≥50 years (N cases = 1833) | 0.94 | (0.90 to 0.98) |  |
| **BMI at 18 years** |  |  | 0.65 |
| <23 kg/m^2^ (N cases = 1855) | 0.98 | (0.94 to 1.01) |  |
| ≥23 kg/m2 (N cases = 499) | 0.91 | (0.84 to 0.98) |  |
| **Smoking** |  |  | 0.79 |
| Never (N cases = 1492) | 0.97 | (0.93 to 1.01) |  |
| Ever (N cases = 877) | 0.95 | (0.90 to 1.00) |  |
| †Adjusted for age, time period of endoscopy, number of reported endoscopies, time in years since most recent endoscopy, reason for current endoscopy, height (inches), body shape at age 5 years (1, 2, 3, 4, 5, ≥ 6), dietary intake during adolescence [from high school FFQ: total calories (quintiles), unprocessed red meat and processed meat (quintiles), total dairy (quintiles), and total fiber (quintiles)], current (adult) aspirin use (≥2 or <2 times/week), current (adult) alcohol intake ( < 4.9, 5–9.9, 10–14.9, 15 + g/d), current (adult) pack-years of smoking (never, 0–10, > 10–20, > 20–40, 40 + pack-years), and time spent watching TV during adolescence (0.5, 0.5–1, 1–1.5, 1.5–2, 2 + h/day). | | | |

| **Table S3**. Joint association of total physical activity during adolescence and adulthood with risk of colorectal adenoma. NHSII 1998-2011 | | | |
| --- | --- | --- | --- |
| **All adenomas** | **Number of cases** | **OR** | **95% CI** |
| low PA adolescence - low PA adulthood | 1192 | 1 |  |
| low PA adolescence - high PA adulthood | 453 | 0.91 | (0.82-1.02) |
| high PA adolescence - low PA adulthood | 463 | 0.93 | (0.83-1.04) |
| high PA adolescence - high PA adulthood | 265 | 0.76 | (0.66-0.88) |
| **Advanced adenomas** |  |  |  |
| low PA adolescence - low PA adulthood | 305 | 1 |  |
| low PA adolescence - high PA adulthood | 105 | 0.87 | (0.69-1.09) |
| high PA adolescence - low PA adulthood | 103 | 0.79 | (0.63-0.99) |
| high PA adolescence - high PA adulthood | 53 | 0.61 | (0.45-0.82) |
| **Non-advanced adenomas** |  |  |  |
| low PA adolescence - low PA adulthood | 659 | 1 | 1 |
| low PA adolescence - high PA adulthood | 258 | 0.93 | (0.80-1.08) |
| high PA adolescence - low PA adulthood | 261 | 0.95 | (0.81-1.10) |
| high PA adolescence - high PA adulthood | 163 | 0.84 | (0.70-1.01) |
| *Adjusted for age, time period of endoscopy, number of reported endoscopies, time in years since most recent endoscopy and reason for current endoscopy. | | | |
| † Additionally adjusted for height (inches), body shape at age 5 years (1, 2, 3, 4, 5, ≥ 6), dietary intake during adolescence [from high school FFQ: total calories (quintiles), unprocessed red meat and processed meat (quintiles), total dairy (quintiles), and total fiber (quintiles)], current (adult) aspirin use (≥2 or <2 times/week), current (adult) alcohol intake ( < 4.9, 5–9.9, 10–14.9, 15 + g/d), current (adult) pack-years of smoking (never, 0–10, > 10–20, > 20–40, 40 + pack-years), and time spent watching TV during adolescence (0.5, 0.5–1, 1–1.5, 1.5–2, 2 + h/day)  High physical activity (PA) at adolescence was defined as the highest tertile (≥53.3 MET-h/week); low was defined as the two bottom tertiles (<53.5 MET-h/week). High PA at adulthood was defined as highest tertile (≥23.1 MET-h/week); low was defined as two bottom tertiles (<23.1 MET-h/week). | | | |
